# The Antinociceptive and Anti-Inflammatory Activities of Caulerpin, a Bisindole Alkaloid Isolated from Seaweeds of the Genus *Caulerpa*

**DOI:** 10.3390/md7040689

**Published:** 2009-11-26

**Authors:** Éverton Tenório de Souza, Daysianne Pereira de Lira, Aline Cavalcanti de Queiroz, Diogo José Costa da Silva, Anansa Bezerra de Aquino, Eliane A. Campessato Mella, Vitor Prates Lorenzo, George Emmanuel C. de Miranda, João Xavier de Araújo-Júnior, Maria Célia de Oliveira Chaves, José Maria Barbosa-Filho, Petrônio Filgueiras de Athayde-Filho, Bárbara Viviana de Oliveira Santos, Magna Suzana Alexandre-Moreira

**Affiliations:** 1 LaFI-Laboratório de Farmacologia e Imunidade, Instituto de Ciências Biológicas e da Saúde, Universidade Federal de Alagoas, Maceió, AL, Brazil; E-Mails: evertontenorio_al@yahoo.com.br (E.T.d.S.); allycq_farmacia@hotmail.com (A.C.d.Q.); difarmacosta@hotmail.com (D.J.C.d.S.); nansa_akino@hotmail.com (A.B.d.A.); eliane_mella@hotmail.com (E.A.C.M.); 2 Laboratório de Tecnologia Farmacêutica, Universidade Federal da Paraíba, João Pessoa, PB, Brazil; E-Mails: daysianneplira@yahoo.com.br (D.P.d.L.); lorenzovitor@hotmail.com (V.P.L.); cchaves@ltf.ufpb.br (M.C.d.O.C.); jbarbosa@ltf.ufpb.br (J.M.B.-F.); athayde_filho@pq.cnpq.br (P.F.d.A.-F.); 3 Laboratório de Algas Marinhas-LAM, Departamento de Sistemática e Ecologia, Universidade Federal da Paraíba, João Pessoa, PB, Brazil; E-Mail: mirandag@dse.ufpb.br (G.E.C.d.M.); 4 Laboratório de Pesquisa em Recursos Naturais, Instituto de Química e Biotecnologia, Universidade Federal de Alagoas, Maceió, AL, Brazil; E-Mail: joaoxjr@yahoo.com.br (J.X.d.A.J.)

**Keywords:** Caulerpa racemosa, antinociceptive, anti-inflammatory, caulerpin

## Abstract

The antinociceptive and anti-inflammatory activity of caulerpin was investigated. This bisindole alkaloid was isolated from the lipoid extract of *Caulerpa racemosa* and its structure was identified by spectroscopic methods, including IR and NMR techniques. The pharmacological assays used were the writhing and the hot plate tests, the formalin-induced pain, the capsaicin-induced ear edema and the carrageenan-induced peritonitis. Caulerpin was given orally at a concentration of 100 μmol/kg. In the abdominal constriction test caulerpin showed reduction in the acetic acid-induced nociception at 0.0945 μmol (0.0103–1.0984) and for dypirone it was 0.0426 μmol (0.0092–0.1972). In the hot plate test *in vivo* the inhibition of nociception by caulerpin (100 μmol/kg, p.o.) was also favorable. This result suggests that this compound exhibits a central activity, without changing the motor activity (seen in the rotarod test). Caulerpin (100 μmol/kg, p.o.) reduced the formalin effects in both phases by 35.4% and 45.6%, respectively. The possible anti-inflammatory activity observed in the second phase in the formalin test of caulerpin (100 μmol/kg, p.o.) was confirmed on the capsaicin-induced ear edema model, where an inhibition of 55.8% was presented. Indeed, it was also observed in the carrageenan-induced peritonitis that caulerpin (100 μmol/kg, p.o.) exhibited anti-inflammatory activity, reducing significantly the number of recruit cells by 48.3%. Pharmacological studies are continuing in order to characterize the mechanism(s) responsible for the antinociceptive and anti-inflammatory actions and also to identify other active principles present in *Caulerpa racemosa*.

## 1. Introduction

Among the many chemical classes present in plant species, alkaloids stand out as one of major importance in the development of new drugs, because they possess a wide variety of chemical structures and have been identified as responsible for many of the pharmacological properties of medicinal plants [1–5]. Some plant families have the genetic capability of producing more than one alkaloid, reflected in the structural diversity of these compounds [6–8].

Indole compounds constitute an extensive family of chemicals found in bacteria, plants and animals. In general, these compounds are related to the metabolism of tryptophan and present substitutions in different positions of the indole ring [6,9]. Indolic compounds with significant and complex physiological roles include those related to melatonin and serotonin (5-hydroxytryptamine). Indole alkaloids possess an indole ring in its structure, constituting a versatile heterocyclic discovery in 1866 [6]; this extensive group of alkaloids received more attention after the isolation of reserpine from *Rauwolfia serpentina* Benth., an alkaloid that changed the history of pathologies conditions as diverse as schizophrenia and hypertension. Indolic alkaloids include various plant-derived medicinal products, including the well-known anti-tumor vinblastine, vincristine, vincamin isolated from *Catharanthus roseus* [10] and camptothecin, is a monoterpene indole alkaloid isolated from the Chinese tree *Camptotheca acuminate* Decne. (Icacinaceae) which displays potent antitumour activity [11].

The knowledge about the chemical composition of marine organisms is an essential element for assessing chemotaxonomic, chemical ecology, and natural products studies, including that directed towards evaluating the pharmacological roles [12–20]. Recently, we initiated a program to investigate the hypothesis that *Caulerpa* species produce secondary metabolites with possible antinociceptive and anti-inflamatory actions. In our preliminary investigation of the crude methanolic extract and phases from *C. racemosa* we had observed an antinociceptive effect in animal models. However, pharmacological and chemical studies are continuing in order to characterize the mechanism(s) responsible(s) for the antinociceptive action and also to identify other active principles present in *Caulerpa racemosa* [21].

**Figure 1.**
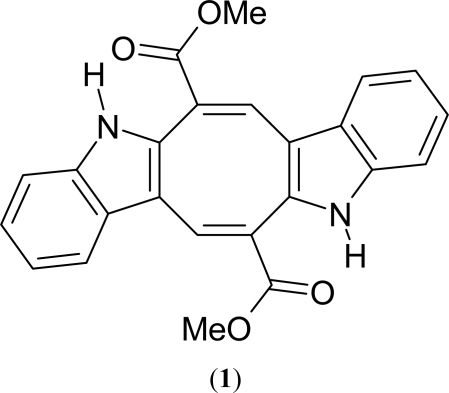
Structure of caulerpin (**1**).

The present study was conducted to characterize caulerpin (**1**, Figure 1), an alkaloid isolated from the lipoid extracts of *Caulerpa racemosa* collected in the Northeast of Brazil. Caulerpin comes from a family of bisindole natural products, and has an extra eight-member ring between the two indole rings which are incorporated directly with the carbonyl group. This alkaloid show a variety of important biological activities already described in the literature, among which it is important to mention the antitumor [22], growth regulator [23] and the plant root growth stimulant properties [24], but its *in vivo* antinociceptive and antiinflamatory activities weren’t reported yet. In addition, caulerpin may also be classified as a compound of low toxicity [25]. However, to date there are a few investigations supporting the pharmacological properties of this seaweed. Thus, this study was intended to evaluate the antinociceptive and anti-inflammatory activities of caulerpin from *Caulerpa racemosa* in animal models.

## 2. Results and Discussion

**Figure 2.**
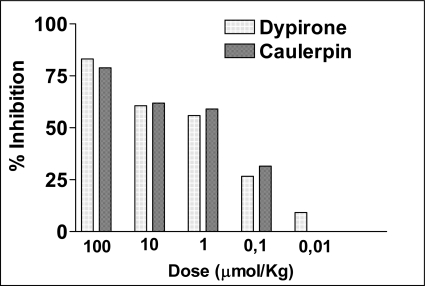
The antinociceptive effect of caulerpin. Dose–response curves of the caulerpin and dypirone (100, 10, 1, 0.1 and 0.01 μmol/kg, p.o.) on the acetic acid-induced writhing model in mice. Each column represents the mean of six animals. Data are expressed as percentage of inhibition of number writhings.

Three different animal models were used in this study to investigate the antinociceptive potential of caulerpin. These methods were selected to ensure both centrally and peripherally mediated effects were investigated. The acetic acid-induced abdominal constriction and the hot-plate methods analyzed peripheral and central activity, respectively, while the formalin test investigated both. In addition, the ear edema induced by capsaicin and the peritonitis induced by carrageenan in mice were used to examine the anti-inflammatory activity of this compound. The acetic acid-induced writhing is a visceral pain model and widely used for the evaluation of peripheral antinociceptive activity [26]. The intraperitoneal administration of an agent that irritates the serous membranes cause a stereotypical behavior in mice which is characterized by abdominal contractions, movements of the body as a whole, twisting of the dorsum abdominal muscles, and a reduction in the motor activity and coordination [27]. The results depicted in Figure 2 shows that caulerpin, given 40 min before, has produced an inhibition of the acetic acid-induced abdominal constrictions in mice. The IC_50_ calculated with 95% confidence interval) for caulerpin was 0.0945 μmol (0.0103–1.0984) (n = 10) and for dypirone it was 0.0426 μmol (0.0092–0.1972) (n = 10). The dates were expressed as % inhibition was compared to the respective acetic acid control group. The mean of contortions of the control group was 42.8 ± 1.9.

Acetic acid causes an increase in the peritoneal fluid level of prostaglandins (PGE2 and PGF2α) as well as lipooxygenase products, involving in part peritoneal receptors and inflammatory pain by inducing capillary permeability [26,28]. Collier *et al.* [29] postulated that acetic acid acts indirectly by inducing the release of endogenous mediators, which stimulate the nociceptive neurons. The most important transmission pathways for inflammatory pain are that comprising peripheral polymodal nociceptors sensitive to protons, such as acid sensitive ion channels and to algogen substances, such as bradykinin and cytokines. Although the writhing test has poor specificity (e.g., anticholinergic, tricyclic antidepressants and antihistaminic and other agents show activity in this test), it is a very sensitive method of screening the antinociceptive effects of compounds [29–31].

In the hot plate test (Table 1), the treatment with morphine (15 μmol/kg, s.c.) caused a marked increase in the latency time of the animals at 60–150 (12.8 ± 0.4 s, 10.3 ± 0.8 s; 9.7 ± 0.7 s; 9.7 ± 0.9 s) and caulerpin (100 μmol/kg, p.o.) caused a marked increase in the latency time of the animals at the times lecture 90–150 (4.6 ± 0.6, 3.8 ± 0.5, 4.0 ± 0.5).

The hot-plate test is commonly used to assess narcotic analgesia [32]. Although the central and peripheral analgesics respond by inhibiting the number of contractions provoked by chemical pain stimuli, only the central analgesics increase the time of response in the hot plate test [33]. These observations tend to suggest that caulerpin may possess centrally- and peripherally-mediated antinociceptive properties. The peripheral antinociceptive effect of caulerpin may be mediated via inhibition of cycloxygenases and/or lipoxygenases (and other inflammatory mediators), while its central antinociceptive action may be due its possible action as partial agonist of adrenergic, serotoninergic, cholinergic and dopaminergic receptors [34,35]. Proposed mechanisms of this effect include interation with serotoninergic receptors, because structural similarity between serotonin and caulerpin [36].

In the present study, administration of caulerpin (p.o.) did not cause motor impairment, as evaluated by forced locomotion in the rotarod test, contrary to diazepam (i.p.) that induced a decrease of the fall latency and increase the number of fall on the rotarod assay. Thus, the possibility that the antinociceptive effect of the compounds tested is due to any degree of motor impairment or sedation is improbable.

Table 2 shows the effects of caulerpin (100 μmol/kg; p.o.) and diazepam (35.1 μmol/kg, i.p.) on the rotarod test from 0.5–1 h. The compound tested did not significantly alter either the fall latency or number of falls when compared with the saline group.

**Figure 3.**
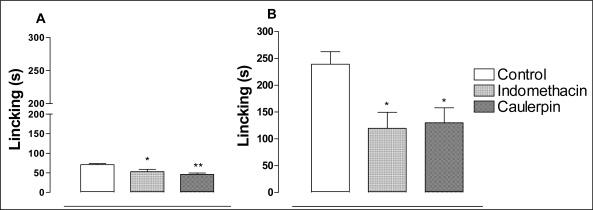
The antinociceptive effect of caulerpin on the formalin test. Caulerpin (100 μmol/kg, p.o.) and indomethacin (100 μmol/kg, p.o.), were assayed in the early-phase (0–5 min, panel A) or late-phase (15–30 min, panel B) of the formalin-induced nociception in mice. Each point represents the mean ± S.E.M. of six animals. Statistical differences between the treated and the control groups were evaluated by ANOVA and Dunnett tests and the asterisks denote the significance levels in comparison with control groups, *P < 0.05,**P < 0.01.

The formalin test in mice confirmed an antinociceptive effect (Figures 3A and 3B). Caulerpin (100 μmol/kg, p.o.) caused a significant inhibition of both neurogenic (28.5%) and inflammatory (55.7%) phases of formalin-induced licking. The treatment with indomethacin (100 μmol/kg, p.o.) was able to inhibit the second phase by 50.3%.

The formalin test is believed to represent a more valid model for clinical pain [37]. The formalin test is a very useful method, not only for assessing antinociceptive drugs, but also helping in the elucidation of the action mechanism. The neurogenic phase is probably a direct result of stimulation in the paw and reflects centrally mediated pain with release of substance P while the late phase is due to the release of histamine, serotonin, bradikynin and prostaglandins [27]. Drugs that act primarily on the central nervous system, such as narcotics, inhibit both phases equally while peripherally acting drugs such as anti-inflammatory non-steroidal (NSAID) and anti-inflammatory steroidal only inhibit the late phase [27,33]. Caulerpin was able to block both phases of the formalin response although the effect was more pronounced in the second phase.

**Figure 4.**
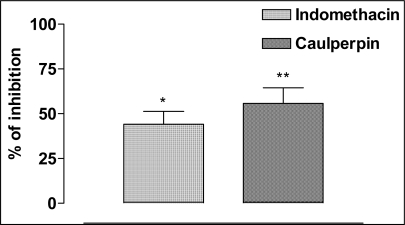
The anti-inflammatory effect of caulerpin. Caulerpin (100 μmol/kg, p.o.) and indomethacin (100 μmol/kg, p.o.) were evaluated on the capsaicin-induced ear edema model. Each column represents the mean ± SEM of five animals. The asterisks denote the significance levels in comparison with control groups, *P < 0.05, **P < 0.01.

To investigate the anti-inflammatory activity we did the model of capsaicin-induced ear edema in mice, a well-characterized and largely used model of inflammation. Topical application of capsaicin was observed to cause a significant rise in plasma extravasations when compared to control ear in the same animal treated with a vehicle. This was significantly reduced by pretreatment with caulerpin, which had shown 55.8% of the inhibition (Figure 4).

The capsaicin-induced ear edema is a classic model used to evaluate the anti-inflammatory activities of compounds [276. Capsaicin (8-methyl-*N*-vanillyl-6-nonenamide), the pungent component of red peppers of the genus *Capsicum*, is a pharmacological tool used to evoke neurogenic acute inflammatory responses such as axon reflex vasodilatation, plasma extravasation, coughing, and painful sensitization [38,39]. The acute neurogenic inflammatory response occurs as a result of neuropeptide release from stimulated primary afferent neurons [40]. Capsacin activates a distinct subpopulation of primary sensory neurons, with somata in the dorsal root, trigeminal as well as nodose ganglia, which are known as transient receptor potential vanilloid 1 (TRPV1). This receptor is a non-selective cation channel expressed in nociceptive fibres that is also activated by heat, protons and some endogenous substances known to be associated with tissue inflammation, particularly lipooxygenase products [41,42].

**Figure 5.**
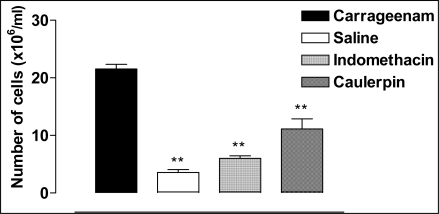
The effect of caulerpin on cell migration. Culerpin (100 μmol/kg, p.o.) and indomethacin (100 μmol/kg, p.o.) were evaluated by the carrageenan-induced peritoneal inflammation test. Each point represents the mean ± S.E.M. of six animals. Statistical differences between the treated and the control groups were evaluated by ANOVA and Dunnett tests and the asterisks denote the significance levels in comparison with control groups, *P < 0.05, **P < 0.01.

In order to evaluate a possible inhibitory effect of caulerpin in cells recruitment into the peritoneal cavity, the carrageenan-induced peritonitis test was used (Figure 5). The positive control group showed increase in the numbers of leukocytes from peritoneal exudates and in the negative control group was not observe augment in the migration of inflammatory cells. Leukocyte cell numbers was significantly reduced up to 4 h after carrageenan injection in peritoneal exudates by indomethacin (72.1%) and caulerpin (48.3%) pretreatments, when compared to exudates from the positive control group (animal inject with carrageenan).

The carrageenan-induced peritonitis test represents a well-characterised model for studies on acute peritoneal inflammation. The action mechanism of carrageenan on peritonitis involves synergism involving prostanoids, leukotriene B_4_ and other chemostactic agents such as C5a and IL-8, which promote an increase of the vasodilatation, plasmatic exudation and recruitment of leukocytes, mainly neutrophils [43].

To confirm the effect of the treatment of caulerpin on recruiting cells, total and differential leukocyte counts were determined in cytospin smears (Table 3). Experimental evidence obtained in this study indicates that caulerpin (100 μmol/kg, p.o.) decreases the neutrophil counts in relation to other leukocytes in peritoneal exudate, suggesting that it is capable of suppresses neutrophil recruitment to the inflammatory sites.

In this test, the results demonstrate that caulerpin reduced significantly the number of recruit cells, showing that this compound presents an anti-inflammatory activity in this cells recruitment model. It is well-known that reactive oxygen species, nitric oxide and PGE_2_ are considered as inflammatory factors, and play important roles in damage of tissues by inflammation [44]. Reactive oxygen species and proteases produced by activated polymorphonuclear neutrophils cause or exacerbate the inflammatory reactions. It has been shown previously that certain prostanoids can inhibit various functions of the neutrophils. It has been suggested that inhibition by endogenously generated prostaglandins is one of the mechanisms by which the pro-inflammatory effects of the neutrophils are limited [45].

Recently, a large number of different kinds of naturally occurring alkaloids with antinociceptive activity have been reported, such as pronuciferine, glaucine, nuciferine and pukateine [46]. Moreover, studies have shown that the alkaloid could present an important anti-inflammatory activity. These compounds can suppress antigen and mitogen-induced lymphocyte proliferation, natural-killer cell cytotoxicity, histamine release by mast cells, interleukin-1 (IL-1) secretion by human monocytes and the action of PAF on platelets [47].

Indole, the potent pharmacophoric nucleus, has been reported to possess a wide variety of biological properties as anti-inflammatory, anticonvulsant, cardiovascular, antibacterial and antifungal [47,48]. Like caulerpin, described in this article, ajmaline, annomontine, cryptolepine, evodiamine and reserpine are examples of other indole alkaloids described in the literature as active in inflammation models [47].

Caulerpin also presents structural similarity with indomethacin, an indole arylacetic acid class nonselective cyclooxygenase (COX) inhibitor and widely prescribed NSAID. In addition to suppressing prostaglandin formation by inhibiting COX, indomethacin is known to possess COX-independent activity such as suppression of malignant transformation of embryonic fibroblasts and activation of the proliferator-activated receptor α and γ [49]. Indomethacin is also a known phospholipase A_2_ inhibitor [50].

The anti-inflammatory activity of caulerpin probably involves an antioxidant effect. Serotonin was once reported as being able to protect biological tissues against radiation injury. This might indicates the capability of serotonin to interacts with or to scavenge reactive oxygen species generated by ionizing radiation and serotonin derivatives also possess great potential as natural antioxidants [51,52]. In addition, tryptophan, an indole amino acid, when exposed to oxidative stress, tryptophyl radical may be generated from tryptophan, with indole nitrogen as the active center. The sensitivity of tryptophan to oxidative stress as well as the radio protective activity of serotonin may be indicative of the importance of the indole moiety in free radical [51]. Moreover, indomethacin, a indole NSAID, is a potent scavengers of reactive oxygen species and inhibit intracellular oxidative activity and exert antioxidant effects in the liposome membrane [53].

The indole group of caulerpin probably is responsible for the antinociceptive and anti-inflammatory activities of this alkaloid. The mechanisms of actions of antinociceptive and anti-inflammatory of this substance can involve the inhibition of COX and antioxidant activity. Moreover, its central antinociceptive action may be due its possible action as a partial agonist of adrenergic, serotoninergic, cholinergic and dopaminergic receptors. However, further studies are really needed to find out the real mechanisms of actions of this compound, and in fact it’s already currently underway to isolate and characterize other active(s) principle(s).

## 3. Conclusions

From the obtained results nociceptive models (the acetic acid-induced writhings and the formalin tests), it was noticed for the first time that caulerpin has showed a considerable antinociceptive and anti-inflammatory activities. *In vivo* inhibition of pain in the hot plate test by this compound was favorable, indicating that caulerpin exhibits central activity. Indeed, it was also observed in the formalin test that this substance exhibited a high anti-inflammatory activity, which was confirmed on the capsaicin-induced ear edema model and the carrageenan-induced peritonitis. However, pharmacological studies are continuing in order to characterize the mechanism(s) responsible for the antinociceptive and anti-inflammatory actions.

## 4. Experimental

### 4.1. Extraction and Isolation

The green alga *Caulerpa racemosa* was collected in the coastal region of Bessa (7o03′52″S/34o49′51″), João Pessoa, Paraíba State, Brazil in April 2008. The specimen was identified by Dr George Emmanuel Cavalcanti de Miranda. A voucher specimen (JPB 13999) has been deposited in the Herbarium Lauro Pires Xavier of the Universidade Federal da Paraíba, Brazil.

The alga was extracted with MeOH at room temperature and the extract was partitioned between H_2_O and hexane, chloroform, ethyl acetate and *n*-butanol. The chloroform fraction was subjected to separation by Sephadex LH-20 column chromatography with a 1:1 CHCl_3_-MeOH solvent system resulting in the isolation of an orange red pigment. Based on its UV, IR and NMR spectral data and chemical properties, it was assigned the structure of caulerpin (**1**).

### 4.2. Biological Activity Tests

#### Drugs and reagents

The following drugs were used in this study: acetic acid and indomethacin (Merck), arabic gum and dipyrone (Sigma Chemical), and morphine sulphate (Dimorf-Cristalia-BR). A solution of formalin 2.5 % was prepared with formaldehyde (Merck) in saline (NaCl 0.9%). The botanical material was used as suspension in arabic gum in all the experiments.

#### Animals

Adult male and female Swiss albino mice (20–35 g) were used in the experiments. They were housed in single-sex cages under a 12-h light:12-h dark cycle in a controlled temperature room (22 ± 2 ºC). They had free access to food and water. Groups of six animals were used in each test group and control animals received vehicle only. The experiments were performed after the approval of the protocol by the local Institutional Ethics Committee - UFAL (N º 006443/2005-78). All experiments were carried out in accordance with the current guidelines for the care of laboratory animals and the ethical guidelines for investigations of experimental pain in conscious animals [54].

#### The Writhing test

The methods described by Koster *et al.* [55] were used with a few modifications. In brief, the selected groups of animals, consisting of six mice per dose of plant compounds or drug, were used in the test. Animals were pretreated with caulerpin (100 μmol/kg, body wt., p.o.), Positive control mice groups received standard analgesics for comparison, dipyrone for 40 min prior to the i.p. injection of acetic acid 0.6% (0.25 mL). The negative control animals did not receive anything (saline) prior to the i.p. injection of acetic acid. Five minutes after the i.p. injection of acetic acid, the number of writhes exhibited by each mouse was counted for 20 min. The antinociceptive activity was expressed as the reduction on the number of abdominal writhing.

#### The Hot-Plate Test

The hot plate test was used to measure response latency according to the method described by Eddy and Leimbach [56], with minor modifications. In these experiments, the hot plate apparatus (Ugo Basile, Model-DS 37) was maintained at 55.5 ± 1 °C. Animals were placed on the heated surface and the time between placement and licking of the paws or jumping was recorded as latency. Latency was recorded for vehicle control groups (10 mL/kg) or pre-treated groups (100 μmol/kg, body wt., p.o.). The test compounds were administered after animal selection on time of 30 min. The selection was made on the basis of the reactivity on the test. Pre-treatment times 0 and 30 min were used for assay adaptation and selection of the animals, respectively. Only mice showing a reaction time within the range of 4–10 sec. were used in this test. The latency of the reaction to nociception was measured at time 0 and then at 30 min intervals up to the 180 min. The botanical materials were administered at 30 min and treatment latencies were recorded at the following time points: 60, 90, 120 and 150 min.

#### The Rotarod Test

The effect of compounds on locomotor performance was tested on the rotarod apparatus as described previously [57]. Twenty-four hours before the experiments, all animals were trained in the rotarod (3.7 cm in diameter, 16 r.p.m) until they could remain in the apparatus for 60 s without falling. On the day of the experiment, mice were treated with caulerpin (100 μmol/kg p.o) and diazepam (35.1 μmol/kg, i.p) and tested in the rotarod from 0.5–1 h after their administration. The latency to fall and the number of fall from the apparatus were recorded with a stopwatch for up to 240 s.

#### Formalin-Induced Pain in Mice

In brief the animals were pretreated with caulerpin 100 μmol/kg, body wt., p.o.). Positive control mice groups received standard anti-inflammatory for comparison, indomethacin for 40 min prior to the i.p. injection of formalin. The formalin test was performed according to the method of Hunskaar and Hole [58]. Briefly, 20 μL of a 2.5% (v/v) solution of formalin in saline were injected into the sub plantar region of the right hind paw and the quantification of the time that the animal spent licking the paw during the first 5 min (first phase) and from 15 to 30 min (second phase) of post-injection time was recorded. The test was performed at room temperature (22–26 ºC) and strictly actions were taken to exclude environmental disturbances (high temperature, noise and excessive movement) that might interfere with the animal’s response [59].

#### Capsaicin-Induced Ear Edema in Mice

The capsaicin-induced ear edema model was performed according to the method of Mantione *et al.* [60]. Forty minutes after injection of caulerpin and indometacin (100 μmol/kg, body wt., p.o.), 250 μg of capsaicin were applied to the inner and other surface of the right ear of the mice. The control group received vehicle only (10 mL/kg, p.o.). The left ear received acetone, delivered in the same manner. Thirty minutes after capsaicin application, the mice were killed and both ears were removed. Circular sections were taken, using a cork borer with a diameter of 6 mm, and weighed. The increase in weight caused by the irritation was measured by subtracting the weight of the untreated left ear section from that of the treated right ear sections. Ear edema was measured as the differences in weight between the treated and the untreated ear. The percent of inhibition was calculated by using (C-T)/C × 100 (%), where C and T indicate non-treated (vehicle) edema and drug-treated edema, respectively.

#### Carrageenan-Induced Peritonitis in Mice

For this series of experiments, the method described by Ferrándiz and Alcaraz [61] was used. Carrageenan (Sigma Aldrich) was freshly prepared (10 mg/mL) in sterile 0.9% w/v saline, and 250 μL were injected i.p., After 4 hours, the animals were killed by cervical dislocation. The peritoneal cavity was washed with 1.5 mL cold PBS, and after a gentle manual massage, the exudate was retrieved and its volume was measured. The number of recruit leukocytes to the peritoneum was counted in a Neubauer chamber and results were expressed as cells × 10^6^/mL. The exudate was collected and used freshly for cell counts and cytospin preparations. The caulerpin (100 μmol/Kg, i.p.), the carrageenan group (arabic gum, p.o.) and the reference drug (indomethacin, 100 μmol/kg, p.o.) were administered 30 min before the carrageenan injection. In the negative control group, animals have just received the same dose of a vehicle (arabic gum, p.o.) 30 min before the saline injection by intraperitoneal route.

### 4.3. Statistical Analysis

The levels of significance between the experimental groups and the control were made using ANOVA in the tutorial Prisma^®^. The values were considered significant when * p < 0.05. The results were expressed as the average ± S.E.M of the average, as indicated in the legends in the figures. The value of ID50 (nonlinear regression analyses and confidence limits) were determined using the GraphPad Prism computer programme (GraphPad^®^ Software Inc.).

## Figures and Tables

**Table 1.** Time-course of caulerpin (100 μmol/kg, p.o.) and morphine (15 μmol/kg, s.c.) on thermal nociception (hot plate).

**Animal Group**	**Dose (μmol/kg)**	**Pretreatment^a^**		**Post-treatment (min)^a^**			
		**0 min**	**30 min**	**60 min**	**90 min**	**120 min**	**150 min**

Control	---	1.4 ± 0.3	2.2 ± 0.6	1.8± 0.2	3.2 ± 0.3	2.8 ± 0.3	2.6 ± 0.5
Morphine	15	6.9 ± 0.4	5.9 ± 0.3	12.8 ± 0.4**	10.3 ± 0.8**	9.7 ± 0.7**	9.7 ± 0.9**
Caulerpin	100	1.9 ± 0.2	2.3 ± 0.6	3.3 ± 0.5	4.6 ± 0.6**	3.8 ± 0.5*	4.0 ± 0.5*

a results represents the mean ± s.e.m. of six animals.

* *P* < 0.05.

** *P* < 0.01.

* p < 0.05 (ANOVA and Dunnett tests were used to evaluate the significance levels in comparison with zero-time).

**Table 2.** Effect of caulerpin (p.o.) and diazepam (i.p.) administration on the latency for the fifth fall and the number of falls from the rotarod test.

**Treatment (μmol/kg)**	**Fall latency (s)**		**Number of falls (s)**	
	**0.5 h**	**1 h**	**0.5 h**	**1 h**

Saline	198.0 ± 28.1	221.0 ± 19.0	0.4 ± 0.3	0.2 ± 0.2
Caulerpin	182.3 ± 37.3	240.0 ± 0	0.9 ± 0.4	0 ± 0
Diazepan	2.7 ± 0.6**	109.9 ± 36.7**	9.2 ± 2.2**	3.9 ± 1.4**

Each value represents the mean ± S.E.M. of the 10 animals in each group.

** p < 0.01 as compared to both control groups. One way ANOVA followed by Newman-Keubs as the *post hoc* test.

**Table 3.** Profile of cellular recruitment of the treatments with caulerpin. Caulerpin (100 μmol/kg, p.o.) and indomethacin (100 μmol/kg, p.o.) were used in the carrageenan-induced (2.5 mg/animal) peritoneal inflammation test. The cell number were determinated by optical microscopic.

**Treatment**	**Percentage of cells (%)**			
	**Polymorphonuclear**	**Lymphocyte**	**Monocyte**	**Basophil**

Saline	15.5	80.0*	1.5	3.0
Carrageenan	34.5	44.0	20.5	1.0
Indomethacin + Carrageenan	7.0*	68.0*	21.0	4.0
Caulerpin + Carrageenan	6.0*	63.0*	31.0*	0.0

Each point represents the % of inhibition of the cell number versus positive control (carrageenan) of six animals. Statistical differences between the treated and the control groups were evaluated by ANOVA and Dunnett tests and the asterisks denote the significance levels in comparison with control groups,

* *P* < 0.05,

** *P* < 0.01.
